# Four In Silico Designed and Validated qPCR Assays to Detect and Discriminate *Tilletia* *indica* and *T. walkeri*, Individually or as a Complex

**DOI:** 10.3390/biology10121295

**Published:** 2021-12-08

**Authors:** Émilie D. Tremblay, Julie Carey, Guillaume J. Bilodeau, Sarah Hambleton

**Affiliations:** 1 Agriculture and Agri-Food Canada (AAFC), 960 Carling Avenue, Ottawa, ON K1A 0C6, Canada; Julie.Carey@agr.gc.ca; 2 Canadian Food Inspection Agency (CFIA), 3851 Fallowfield Road, Ottawa, ON K2H 8P9, Canada; Guillaume.Bilodeau@inspection.gc.ca

**Keywords:** wheat, ryegrass, Karnal bunt, dwarf bunt, phytopathogen, qPCR

## Abstract

**Simple Summary:**

Plant pathogens represent a constant threat to human and animal food, as well as the economy. International trading is constantly expanding and has been known as a means of transportation and introduction for plant pests (e.g., bacteria, viruses, fungi, and insects) in new areas. They can damage or completely ruin a harvest and there are often strict regulations for the most unwanted plant pests in order to keep their incidence confined. The fungal plant pathogen *Tilletia indica* causes Karnal bunt, a wheat disease that breaks or hollows grains, grows in dark powdery masses, and emits a foul fishy odor, and is therefore highly regulated by a number of country authorities, many of which respond by imposing quarantine regulations. While there are many diagnostic methods developed (microscopy, molecular assays, etc.) to identify Karnal bunt, they have limitations. This study presents four highly sensitive quantitative PCR assays with molecular probes targeting unknown genomic regions for the detection and identification of *T. indica* and *T.* *walkeri*—its closest relative—and the species-complex including both species. Bioinformatics analyses of DNA sequences were used to design the toolkit presented.

**Abstract:**

Several fungi classified in the genus *Tilletia* are well-known to infect grass species including wheat (*Triticum*). *Tilletia indica* is a highly unwanted wheat pathogen causing Karnal bunt, subject to quarantine regulations in many countries. Historically, suspected Karnal bunt infections were identified by morphology, a labour-intensive process to rule out other tuberculate-spored species that may be found as contaminants in grain shipments, and the closely-related pathogen *T. walkeri* on ryegrass (*Lolium*). Molecular biology advances have brought numerous detection tools to discriminate *Tilletia* congeners (PCR, qPCR, etc.). While those tests may help to identify *T. indica* more rapidly, they share weaknesses of targeting insufficiently variable markers or lacking sensitivity in a zero-tolerance context. A recent approach used comparative genomics to identify unique regions within target species, and qPCR assays were designed in silico. This study validated four qPCR tests based on single-copy genomic regions and with highly sensitive limits of detection (~200 fg), two to detect *T. indica* and *T. walkeri* separately, and two newly designed, targeting both species as a complex. The assays were challenged with reference DNA of the targets, their close relatives, other crop pathogens, the wheat host, and environmental specimens, ensuring a high level of specificity for accurate discrimination.

## 1. Introduction

*Tilletia indica* Mitra [1], the causal agent of Karnal bunt on wheat (*Triticum aestivum* and *T. durum*) and triticale (×*Triticosecale*) is considered a serious threat to crop production in many regions [2,3]. The fungal disease was initially found in Karnal, India, in 1931, and currently occurs mainly in Asian countries such as India, Afghanistan, Iran, Iraq, Pakistan, and Nepal [1,4,5,6,7]. The first detection in North America was in 1972 in Mexico, and it has since been reported in Brazil, South Africa, and USA [4,5,8,9,10,11,12]. Some authors claim that the importance drawn to it is overrated given its limited distribution [13,14], yet many countries (Canada, USA, Mexico, New Zealand, Morocco, Israel, Belarus, Norway, and other European countries) continue to impose quarantine pest regulations on wheat imports and exports [4,5] to prevent further spread. The pathogen can be dispersed quickly and is challenging to eradicate once its spores are present in soil, where they can survive for up to five years [5,9,15,16]. The spread of Karnal bunt spores is likely to cause infection, which can dramatically hamper a harvest and reduce production due to kernel bunting, and the foul smell and taste associated with the infection renders wheat and wheat-products such as flour unfit for consumption [4,17,18,19,20,21].

The detection of Karnal bunt in the USA in 1996 quickly escalated into an embargo on shipments because of the scale of this country’s wheat-exports [22,23]. Many American and European countries free from Karnal bunt established a ban on imports of wheat, triticale, and other susceptible crops from affected countries or imposed mandatory phytosanitary certification for imported grain to be free from *T. indica* and to originate from pest-free states or areas [24,25]. The USA also went through a country-wide quarantine survey program to screen for and contain outbreaks in affected areas [26]. Although it is reported that seed-borne diseases can be controlled using fungicides or chemical treatments, there is also a recent upsurge in those diseases due to biological agriculture practices [27,28,29]. In addition, it is known that climate change could enhance the emergence of plant pathogens such as Karnal bunt given the occurrence of proper temperature and moisture conditions newly met by certain wheat growing areas [30,31,32,33].

It is essential to have the resources to differentiate *T. indica* from morphologically and genetically similar species given the social, economical, and environmental impacts at stake [34]. Rapid and sensitive tools to detect and identify *Tilletia* species are required for Karnal bunt management and forecasting, especially for the leading countries of wheat exports which, in 2018, were Russia (US $8.4 billion/year), Canada (US $5.7 billion/year), and USA (US $5.4 billion/year) [35]. Although there are over a hundred *Tilletia* species, most do not infect wheat and have little to no impact for crop production. Amongst the species closely-related to *T. indica*, the ryegrass pathogen *T. walkeri* Castlebury and Carris [23], as yet reported only from Australia, China, New Zealand and USA [36], is of concern as a grain contaminant. The species has received the attention of numerous scientists attempting to decipher minor, yet critical morphological or molecular differences between the two congeners [4,12,13,14,22,23,37,38,39,40]. Ryegrass is also commonly cultivated in wheat-growing regions, increasing the risk of mixtures in seed lots [23,41].

Several molecular assays allowing researchers to discriminate or detect selected *Tilletia* spp. are already available (Table 1), although many of them have limitations. For instance, as previously reported [42,43,44], the Internal Transcribed Spacer (ITS) region is a marker with limited variation between *T. indica* and its closest relative, *T. walkeri*. This region has been exploited by approaches such as Random Amplified Polymorphic DNA (RAPD) combined with Polymerase Chain Reaction (PCR) and Restriction Fragment Length Polymorphism (RFLP) [45], PCRs or PCR-combined methods [44,46,47,48], RFLP [42], and real-time PCR (qPCR) [49,50,51]. There are only two consistent single nucleotide polymorphisms (SNPs) between the publicly available ITS sequences of the two species [42,43,44]. All but one of the diagnostic assays sanctioned by the International Plant Protection Convention (ISPM 27) [52] for *T. indica* diagnostics are based on ITS [44,45,49], while the assay by Frederick et al. [37] targets mitochondrial DNA.

Recognized in the scientific community studying *Tilletia* spp., the ITS-related limitation was addressed by scientists who thereafter focused on alternate regions such as mitochondrial DNA. While PCR assays were designed to differentiate the causal agent of Karnal bunt from other smut fungi or *Tilletia* species [46,53,54], a qPCR assay was developed to distinguish *T. indica* and *T. walkeri* [37]. Two Loop-mediated isothermal Amplification (LAMP) assays were also designed to differentiate *T. indica* from other close relatives [16,55]. However, Tan et al. [16] reported that the LAMP assay by Gao et al. [55] had specificity issues with some *T. indica* isolates. An assay was also designed in an unknown region by Mishra et al. [56]. One limitation shared by all of those non-ITS targeting assays is their level of sensitivity, which is critical in an absolute absence requirement context such as for Karnal bunt. The qPCR assay of Frederick et al. [37] could detect 5 pg of DNA, making it twice as sensitive as the LAMP assays of Gao et al. [55] at ≥10 pg of DNA or Tan et al. [16] at 10 pg of fungal DNA. Plus, although the assay by Ferreira et al. [54] is more sensitive (1 pg), it cannot discriminate *T. walkeri*.

Other limitations include the inability to detect more than one species simultaneously or a requirement for teliospore germination prior to molecular analysis, which slows the diagnostic process. Several previously developed assays [42,53,56] face one or both, rendering them fairly low-throughput. The international protocols for the diagnostic of *T. indica* involve morphological observations, isolation, and germination of single spores [52], which are time-consuming and require highly-trained personnel, in addition to molecular assays targeting the ITS region [44,45,49] or mitochondrial DNA [37]. Similarly, approaches like size-selective sieving of teliospores, a modified version of the general seed-wash centrifuge method, can be limited by the low number of spores present or the low germination frequency related to dormancy [15,50,57,58].

Different approaches have recently been taken to attempt a better differentiation method for *T. indica* and its close relatives. For instance, Sharma et al. [59] screened for simple sequence repeats, or microsatellites, for diagnostics and genetic diversity studies of smut and bunt fungi and they included cross-transferable markers for *T. indica*. Promisingly, the project appears to be the first one to develop microsatellites for identification and validation of *T. indica*. Given sufficient levels of polymorphism across genera, the tool has great potential to evaluate genetic variation, but it requires further testing for a more comprehensive validation. Nguyen et al. [43] took a comparative genomics bioinformatics approach to screen for signature, unique, and single-copy regions theoretically variable enough to differentiate all the unwanted wheat-infecting *Tilletia* species; i.e., *T. caries*, *T. controversa*, and *T. laevis* as well as *T. indica*. Although their methods identified candidate regions, their High-Throughput/WGS protocol was only tested in silico.

The objective of this study was to perform wet-laboratory validation and optimization of the *T. indica* and *T. walkeri* candidate assays from Nguyen et al. [43] and design additional new assays for detecting both species at once. Extensive testing was performed using reference materials and environmental specimens to assess specificity and sensitivity. The result is an array of four qPCR assays that can determine whether samples—e.g., field-collected specimens, pure cultures, or seed lots—comprise entities from the *T. indica*/*T. walkeri* complex and if so, identify whether either or both are present, at a highly sensitive detection level.

## 2. Materials and Methods

### 2.1. Fungal Material and DNA Extraction

Forty-eight pure culture isolates representing 11 *Tilletia* species were obtained to serve as reference material for this study (Table 2), including 20 for *T. indica* and 3 for *T. walkeri*, the 2 target species. The Canadian Collection of Fungal Cultures, Ottawa, Canada (DAOMC) strains were cultured as polysporidial isolates from surface-sterilized germinated teliospores by the Canadian Food Inspection Agency (CFIA) and most were included in a study by McDonald et al. [47]. They were later provided to Agriculture and Agri-Food Canada (AAFC) as pure cultures for research purposes and for long term preservation in DAOMC. The rest were obtained from the American Type Culture Collection (ATCC; Manassas, VA, USA) or the CBS-KNAW Filamentous Fungi Collection (CBS, Westerdijk Fungal Biodiversity Institute, Utrecht, Netherlands). DNA was extracted from cultures grown on solid potato dextrose agar (Difco, Becton Dickinson, Franklin Lakes, NJ, USA) at room temperature in the dark and using one of the following kits with the manufacturer’s instructions: CTAB (https://www.protocols.io/view/fungal-ctab-dna-extraction-bhx8j7rw, accessed on 8 November 2021), DNeasy Plant Mini kit (QI, Toronto, ON, Canada), E.Z.N.A.^®^ Fungal DNA Miniprep kit (VWR, Mississauga, ON, Canada), Macherey-Nagel Nucleospin^®^ 96 Plant or Macherey-Nagel NucleoMag^®^ 96 Trace kit (Macherey Nagel GmbH & Co. KG, Düren, Germany), OmniPrep^TM^ for Fungi kit (G-Biosciences, St. Louis, MO, USA), or UltraClean^TM^ Microbial DNA Isolation Kit (MO BIO Laboratories Inc., Carlsbad, CA, USA).

Environmental specimens consisting of dried plant parts or seeds infected with various *Tilletia* species were obtained from collaborators at the United States Department of Agriculture (Table 3). For each specimen, teliospores from a single spore ball, or for *T. pallida* from multiple seeds, were sampled and DNA was extracted with the Nucleomag 96 Trace Kit on a Kingfisher mL automated system (ThermoFisher Scientific, Waltham, MA, USA), with the following customizations. Prior to extraction, samples were ground using liquid nitrogen and sterile disposable micro-centrifuge tube pestles (PES-15-B-SI, Axygen, Corning, NY, USA) or homogenized using a Bertin Precellys 24 tissue homogenizer instrument (Bertin Technologies SAS, Montigny-le-Bretonneux, France) set at 6000 rpm for one cycle of 40 s. Tubes containing samples and 200 µL FLB were gently vortexed for 15 s, spun at 1500× *g* for 15 s, incubated at 56 °C for 30 min (to ensure optimal lysis) while being mixed by flicking occasionally. Next, 30 µL of RNase Cocktail Enzyme Mix (ThermoFisher Scientific) solution and 10 µL of RNase A (20 mg/mL) (ThermoFisher Scientific) were added (to reduce the amount of RNA), and the incubation, vortexing, mixing steps repeated. Twenty-five microliters of the proteinase K solution were added to each sample, with vortexing and mixing repeated. Then, samples were incubated for 1 h at 56 °C, centrifuged at 5600× *g* for 5 min and the supernatant of the lysed samples (≥225 µL) transferred to the Kingfisher mL machine for processing.

Identifications and success of all DNA extractions were confirmed by ITS sequencing using primers ITS5 and ITS4 [61] or ITS5 and LR5 or LR6 [62] for a longer fragment, which includes a portion of the 28S region. Methods for PCR and Sanger sequencing were as described in Malloch et al. [63] for DNA from pure cultures. For some of the dried environmental specimens, sequencing with these primer combinations failed, so *Tilletia*-specific primers MK56-F and Tilletia-R [44]—targeting the ITS1 only—were used, with similar PCR and sequencing protocols except for the following modifications. Bovine Serum Albumin (20 mg/mL) (ThermoFisher Scientific) was added to the master mix, with a corresponding reduction of H_2_O, to enhance PCR success and 45 cycles run instead of 40. Sequences were edited using Geneious Prime 2021.2.2 (https://www.geneious.com, accessed on 8 November 2021).

### 2.2. Species-Specific TaqMan qPCR Assay Validation for T. indica and T. walkeri

Using a comparative genomics approach combined with bioinformatics analyses to identify single-copy orthologous genes unique to the species targeted, Nguyen et al. [43] developed in silico designed qPCR primers and probes for specific detection of four *Tilletia* species, but no wet-laboratory testing was performed. In this study, preliminary testing using small test sets of reference DNA extracts determined that the one assay targeting *T. indica* (OG09272) and one of the three assays targeting *T. walkeri* (OG10415) from that study warranted further validation (Table 4). Both of these species-specific qPCR assays were optimized and tested at the CFIA Ottawa Laboratory Fallowfield (CFIA-OLF) against the complete set of reference target and non-target strains or specimens (Table 2 and Table 3; Supplementary Table S1).

### 2.3. Complex-Specific TaqMan qPCR Assay Design

Following the orthologous genes approach used by Nguyen et al. [43], searches were performed to identify additional gene regions suitable for development of assays that are (1) specific to the complex of both target species—which form a monophyletic clade within the genus [43,60,64]—and (2) diagnostic against the other species known to occur on wheat. The 10 genome assemblies and annotations published by Nguyen et al. [43] for strains of *T. indica* (x3), *T. walkeri* (x2), *T. caries* (x1), *T. controversa* (x2), and *T. laevis* (x2) were retrieved from the NCBI database (Table 5).

The orthologous groups defined and the phylogenomics analyses performed in that study using OrthoFinder v1.1.8 [65] were obtained from the authors. New searches identified a candidate single-copy gene common to all five species but distinct for the two targets as compared to the other species (OG01193). Then, using Geneious Prime (2020.0.5) (https://www.geneious.com, accessed on 8 November 2021) and the target genome assemblies, a sequence comparison approach consisting of visual screening for regions of interest (i.e., with suitable length and variability for the primers and probe) was used to identify a second candidate gene that was common only to both target species (OG08220). Primers and TaqMan probes were designed for each new gene region (Table 4).

### 2.4. End-Point PCR Primer Testing

The species-specific qPCR assays named here as TwaOG10415 and TinOG09272 were tested at 60 °C, 58 °C, and 56 °C based on the primers’ annealing temperature recommended in Nguyen et al. [43] and then optimized parameters were selected accordingly. The Eco Master Mix (ThermoFisher Scientific; Cat# A41141, custom order, on request) was used. Similarly, the primers designed for the complex-specific assays (i.e., OG01193 and OG08220) were challenged and optimized prior to performing real-time PCR tests. The optimum primer annealing temperatures were determined by PCR using temperature gradients of 52–60 °C and then 61–70 °C, where increments were automatically determined by the Eppendorf Mastercyler pro S instrument (Eppendorf, Hambourg, Germany). The Titanium Taq DNA polymerase (Takara Bio Inc., Nojihigashi, Kusatsu-shi, Shiga, Japan) was used for the initial tests to maximize the amplification success considering that this enzyme can be more permissive than others due to its high robustness conferring high-yield PCR [66]. Given an observed failure of amplification at the higher range of potentially optimal temperatures using the Eco Master Mix (data not shown), all additional tests were run using the QuantiTect Probe PCR Kit (QIAGEN, Hilden, Germany). Details on the PCR reactions volumes, parameters, master mixes tested, and electrophoreses can be found in Appendix A.

### 2.5. Real-Time PCR Specificity and Detection Limit

The assessment of specificity for all four candidate assays was performed in three steps: (1) initially against small test sets of target strains and the non-target species *T. controversa* DAOMC 236426, followed by (2) testing against the complete set of DNA samples, including *T. indica* (x31), *T. walkeri* (x3) and (3) all other reference strains, environmental specimens, and a diverse set of non-*Tilletia* non-targets (Table 2 and Table 3, Supplementary Table S1). All samples and negative water controls were run in triplicate for all four assays. DNA extracts were quantified using a Qubit 2.0 fluorometer (ThermoFisher Scientific) and normalized prior to final qPCR validation testing. Both complex-level assays (i.e., OG01193 and OG08220) were tested at the AAFC Ottawa Research and Development Center (AAFC-ORDC) and consisted of a 10 µL reaction of 0.4 µM of each forward and reverse primer, 0.1 µM of the TaqMan probe, and one unit of 2 × QuantiTect Probe PCR Kit. The 2-step cycling conditions, run on a LightCycler 480 Instrument II (Roche, Basel, Switzerland), were 15 min at 95 °C followed by 50 cycles of 15 s at 95 °C and 1 min at 68 °C (OG01193) or 60 °C (OG08220). Both 3-step species-level assays (i.e., TwaOG10415 and TinOG09272), tested at the CFIA-OLF, consisted of a 25 µL reaction of 0.48 µM of each forward and reverse primer, 0.025 µM of TaqMan probe, and one unit of 2 × TaqMan Eco Master Mix. The cycling conditions, run on a ViiA 7 Real-Time PCR System (ThermoFisher Scientific), were 5 min at 95 °C followed by 50 cycles consisting of 15 s at 95 °C, 30 s at 56 °C and 72 °C for 30 s.

The limit of detection (LOD) for each assay was assessed with reference DNA for the target species, *T. indica* DAOM 236416 and *T. walkeri* DAOMC 236422 using six serial dilutions (1:10) of DNA normalized to approximately 2 ng/uL (i.e., 2.2 to 2.2 × 10^−6^ ng/µL). Standard curves for the two species-specific assays were assessed at CFIA-OLF and AAFC-ORDC, on two different instruments. Those for the complex assays, for both target species, were completed at AAFC-ORDC only. To robustly evaluate the LOD, 15 additional replicates for three of the lowest concentrations (i.e., 2.2 × 10^−3^ to 2.2 × 10^−5^ ng/µL) were tested for each assay.

Challenging assay specificity was done by running each one against DNA from 35 strains or environmental specimens representing 10 non-target *Tilletia* species, namely *T. asperifolia*, *T. brevifaciens*, *T. bromi* (= *T. bromi-tectorum*), *T. caries*, *T. controversa*, *T. fusca*, *T. goloskokovii*, *T. horrida*, *T. laevis,* and *T. pallida* (Table 2 and Table 3). In addition, the four assays were tested against DNA from an uninfected wheat host plant and from 25 strains or herbarium specimens representing 19 non-*Tilletia* wheat and/or grain-crop pathogens causing rusts (*Puccinia* spp.), smuts (*Ustilago nuda* and *Urocystis tritici*), molds (*Cladosporium* spp., *Aspergillus foetidus* and *Penicillium verrucosum*), spots and blights (*Didymella glomerata*, *Pyrenophora tritici-repentis, Septoria glycines* and *Fusarium graminearum*), blotches (*Parastagonospora nodorum* and *Bipolaris sorokiniana),* powdery mildew (*Blumeria graminis*), and black point and smudge (*Alternaria alternata*) (Supplementary Table S1).

### 2.6. Test Validation of The IPPC Sanctioned ITS1 qPCR Assay

Using the same reference DNA extracts and serial dilutions as were used to generate standard curves in Section 2.5, the qPCR assays published by Tan et al. [49] for *T. indica*, *T. walkeri,* and *T. horrida* were tested at AAFC-ORDC individually and also as a three-plex assay, as validated by Valente et al. [50], following the same protocol. The qPCR reaction mix components and cycling conditions were not changed from the original reference. For these tests, the reference DNA was normalized to ~1 ng/µL before making 10-fold serial dilutions.

## 3. Results

### 3.1. Fungal Material and DNA Extraction

An ITS DNA barcode sequence was generated for each of the reference fungal cultures or specimens in this study, using DNA concentrations normalized at 1 ng/µL, and deposited in GenBank (Table 2 and Table 3; Supplementary Table S1). For most, the complete ITS sequence was determined. For some of the field-collected environmental specimens, the use of group-specific primers that amplify the ITS1 region only were required to avoid amplification of the host or contaminants. Identifications were verified by sequence alignments (BLAST searches) on NCBI and by DNA sequence analyses using Geneious (data not shown).

### 3.2. Species-Specific and Complex-Specific qPCR Assay Design

The primers and probes for the two assays designed by Nguyen et al. [43] and the two complex assays designed in this study are listed in Table 4. Sequence alignments of *T. walkeri* and *T. indica* showing the gene regions used to design qPCR assays OG08220 and OG01193, and the primer and probe locations, are presented in Supplementary Data S1.

### 3.3. End-Point PCR Primer Testing

For the species-specific assays TwaOG10415 and TinOG09272, primer testing at temperatures over 56 °C and using 2-step reactions revealed critical problems that translated into large DNA smears when visualised on the Qiaxcel instrument (data not shown). The results from the optimized 3-step reactions (i.e., extension step added, and decreased annealing time) at 56 °C displayed a single clear band on gel. Results from the temperature gradient testing for the primers of the two complex-specific qPCR assays OG01193 and OG08220 were used to determine the annealing temperature to be subsequently used for qPCR. Using the QuantiTect Probe PCR mix, OG01193 showed single target-sized bands (≈150 bp) at 68.5 °C on gel, whereas OG08220 displayed similar results over a broader temperature range of 58.5–68.5 °C. Based on the additional qPCR tests (data not shown), the respective annealing temperatures picked for downstream qPCR proceeding were 68 °C and 60 °C.

### 3.4. Real-Time PCR Specificity and Detection Limit

**Figure 1:**
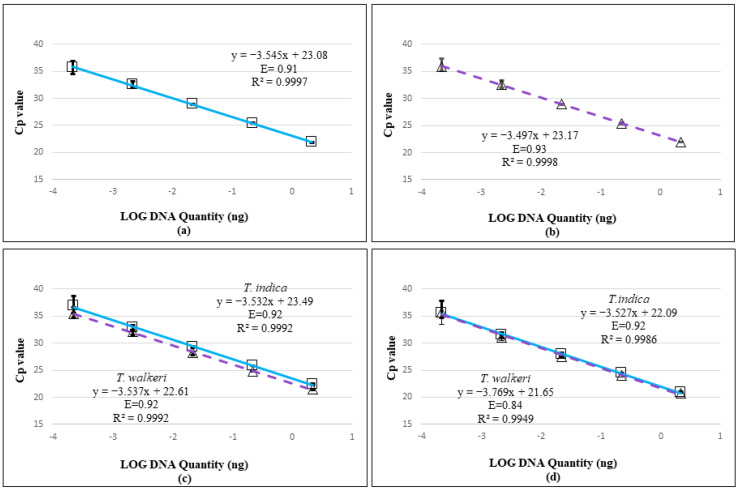
Standard curves showing the regression between DNA log quantities (ng, x-axis) and cycle thresholds (Cp, y-axis) for the four qPCR assays, generated with serial dilutions (~2.2 to ~2.2 × 10^−4^ ng/µL) of the target species, *T. indica* DAOMC 236416 (squares/solid line) and *T. walkeri* DAOMC 236422 (triangles/dotted line). (**a**) TinOG09272 specific to *T. indica*; (**b**) TwaOG10415 specific to *T. walkeri*; and complex-specific detecting both species, (**c**) OG08220 and (**d**) OG01193. Plotted are the average Cp values for the initial 3 replicates run for each dilution and error bars for all replicates (3 for the first three dilutions, 18 for the last 2).

Testing against the complete panel of DNA extracts (Table 2 and Table 3; Supplementary Table S1) confirmed the specificity of the four new qPCR assays. All *T. indica* and *T. walkeri* reference strains and environmental specimens were positively detected and all non-target *Tilletia* species, other fungi, and the wheat host were negative. For all four assays and for both target species, the standard curves amplified consistently up to the fourth dilution, i.e., ~2.2 × 10^−4^ ng/µL (Figure 1), indicating an LOD of 0.22 pg (=220 fg). Results at CFIA-OLF for the species-specific assays (data not shown) were consistent with results at AAFC-ORDC. The actual LOD was determined to be between 0.22 pg and 0.02 pg because some replicates also amplified at the 10^−5^ dilution: 22% for both species-specific assays, 17% for OG08220 and 39% for OG01193 with *T. indica*, and 44% for OG08220 and 33% for OG01193 with *T. walkeri*. Amplification efficiencies for each assay with each target are shown on Figure 1.

### 3.5. Test Validation of The IPPC Sanctioned ITS1 qPCR Assay

Three of the assays included in the Tan et al. [49] 5-plex set of ITS assays were validated on our LightCycler 480 instrument using our reference DNA samples and they performed as expected. Our standard curve tests confirmed LODs close to 0.1 pg for both the *T. indica* and *T. walkeri* assays (data not shown). Although cross-reaction of the two species was observed, the amplification curves were different and by using the recommended allelic discrimination step at the end of each run, both species were successfully differentiated. The *T. horrida* assay confirmed identifications of three sampled environmental specimens (Table 3) and was negative for both *T. indica* and *T. walkeri*.

## 4. Discussion

The qPCR assays validated in this study were developed for single-copy genomic DNA regions of the target species and to provide new tools to identify and discriminate the closely-related phytopathogens *T. indica* and *T. walkeri*. The former species is of high concern and is subject to regulations related to imports and exports of wheat and grain shipments. The latter, a ryegrass pathogen, presents challenges for accurate differentiation from *T. indica* because of similarities in teliospore morphology and limited ITS sequence differences, a multi-copy gene region commonly used for fungal identifications and as a basis for DNA-based assay development.

The experimental LOD obtained is similar across all four assays, approximately 200 fg, which is highly sensitive for single copy genes and more sensitive than several published molecular assays targeting mitochondrial DNA (= multi-copy) such as those by Tan et al. [16], Frederick et al. [37], and Gao et al. [55]. By comparison, Bilodeau et al. [67] obtained a detection of 3 fg for a qPCR assay targeting the Intergenic region in *Verticillium* species, which was estimated to be ~24 to 73 copies per haploid genome, with an average of ~46 copies depending on the isolate. It can be inferred that the estimate for a single copy gene would be between 200 and 75 fg, similar to the actual LOD for each assay in this study, which is between 200 and 20 fg. This compares favourably with published ITS qPCR assays, such as those with TaqMan probes published by Tan et al. [49] and recommended by the IPPC [52], reported to be 0.1 pg (=100 fg). Gurjar et al. [51] published an ITS qPCR SYBR Green assay with an LOD of 0.1 pg, but it was not validated against samples of *T. walkeri*. Assays based on ITS have the advantage of targeting a multi-copy gene, hence the lower LODs, but their specificity is based on a single SNP either in the ITS1 [49] or ITS2 [51]—non-coding regions subject to mutation—and depends on discrimination at the allelic level for accurate diagnostics of *T. indica* and *T. walkeri*.

Besides the comprehensive testing using reference DNA for multiple *Tilletia* species, our assays were also challenged with DNA from environmental specimens and a broad range of non-target fungi that also occur on wheat, demonstrating robustness. Testing was completed in two different laboratories using different instruments for the species-specific versus complex assays, while the standard curve validations were all completed by the same laboratory and machine, demonstrating transferability. The level of resolution achieved is, in part, attributable to the genome-wide in silico approach that revealed the unknown genomic regions used here, instead of the insufficiently variable ITS. Nguyen et al. [43] reported a pronounced difference in estimated genome size between the two target species—approximately 30 Mb for *T. indica* and about 24 Mb for *T. walkeri*—which facilitated the discovery of the unique regions targeted for three of our four assays. By contrast, the OG01193 locus is common to all four species sequenced by Nguyen et al. [43] but has marked sequence variation between the targets’ complex and the *T. caries*/*T. controversa*/*T. laevis* complex (Supplementary Data S1).

## 5. Conclusions

The new assays presented here offer an efficient, high-throughput and directly usable tool for the detection of Karnal bunt and ryegrass bunt from infected material, while allowing diagnostic labs to reduce their reliance on time- and resource-consuming pre-treatments and analyses such as microscopy, single-spore isolation, teliospore germination and pure culture isolations. Complex and species-specific identification is possible using three of them. The assay pairs provide a hierarchical approach to diagnostics, whereby either of the two that target the species complex (i.e., OG08820 and OG01193), or both for more robust confirmation, can be used as a preliminary test to assess the presence/absence of either species. If positive results occur, additional testing can follow using the two assays that target unique genomic regions (i.e., TinOG09272 and TwaOG10415) for species-specific detection. This approach can be used in combination with spore identification and quantification using microscopy of grain or seed wash samples, providing additional evidence for regulatory decision-making and increasing sample processing throughput.

While there is more work to be done to enhance the discrimination of other highly unwanted species such as *Tilletia controversa*, the causal agent of dwarf bunt, the combination of bioinformatics, and molecular biology technologies used here certainly should be considered as a faster way of screening for key regions within genomes. *Tilletia controversa* is, like *T. indica*, another pathogen that has several closely-related species (including the common bunt species *T. caries* and *T. laevis*) that are generally less concerning for wheat production and international trade, hence the importance of achieving high resolution and sensitivity for diagnostics.

## Figures and Tables

**Table 1:** Chronological summary of assays developed to detect or discriminate *Tilletia* species.

Year	Type of Assay	Purpose of Assay	References
***Targeting ITS ^a^ region***			
1998	RAPD ^b^ + PCR ^c^ + RFLP ^d^	Discriminate *T. indica* from other *Tilletia* species	[45]
2000	REP ^e^-PCR genomic fingerprinting	Separate species from the *T. indica*/*T. walkeri* complex from those of the *T. controversa* complex ^f^	[47]
2001	RFLP	Discriminate *T. indica* from *T. walkeri*	[42]
2006	2 step PCR + FRET ^g^	Discriminate *T. indica* from *T. walkeri*	[44]
2006	PCR + dot blot	Discriminate *T. caries*, *T. foetida* and *T. controversa*	[48]
2009	5-plex qPCR ^h^	Discriminate *T. indica, T. walkeri, T. horrida, T. ehrhartae* and the *T. controversa* (broad range) complex	[49]
2011	PCR	Discriminate *T. indica* from *T. horrida* and *T. caries*	[46]
2017	qPCR	Detect spores of *T. indica* in soil	[51]
2019	5-plex qPCR	Validation of Tan et al. 2009 qPCR assays	[50]
***Targeting Mitochondrial DNA***			
1996	PCR	Discriminate *T. indica* from other smut fungi	[53]
1996	PCR	Discriminate *T. indica* from other *Tilletia* species	[54]
2000	qPCR	One primer set to detect *T. indica,* one primer set to detect *T. walkeri*	[37]
2011	PCR	Discriminate *T. indica* from *T. horrida*	[46]
2016	LAMP ^i^	Discriminate *T. indica* from other closely-related species	[55]
2016	LAMP	Discriminate *T. indica* from other closely-related species	[16]
***Targeting unknown region***			
2002	RAPD-PCR	Discriminate *T. indica* and *T. barclayana*	[56]

^a^ Internal Transcribed Spacer. Assays may target the whole region or solely part of it; ^b^ Random Amplified Polymorphic DNA; ^c^ Polymerase Chain Reaction; ^d^ Restriction Fragment Length Polymorphism; ^e^ Repetitive-Sequence-Based; ^f^ Stated in the publication as including *Tilletia caries*, *T. laevis*, *T. contraversa*, *T. fusca*, *T. bromi* and *T. goloskokovi*; ^g^ Fluorescence Resonance Energy Transfer; ^h^ Quantitative Polymerase Chain Reaction; ^i^ Loop-Mediated Isothermal Amplification.

**Table 2:** Voucher numbers, host genus, provenance, year collected, ITS GenBank accession numbers and assay validation results for the reference *Tilletia* strains used in this study.

Species	Voucher No. ^a^	Host Genus	Year Collected	Provenance	ITS GenBank Accession No.	TaqMan qPCR Results			
						TinOG09272 *T. indica*	TwaOG10415 *T. walkeri*	OG08220 *T. indica* and *T. walkeri*	OG01193 *T. indica* and *T. walkeri*
*T. indica*	DAOMC 236406	*Triticum*	1996	Mexico	OL653674	+	−	+	+
	DAOMC 236407	*Triticum*	1995	India	OL653675	+	−	+	+
	DAOMC 236408	*Triticum*	1997	India	OL653676	+	−	+	+
	DAOMC 236409	*Triticum*	1997	India	HQ317520	+	−	+	+
	DAOMC 236410	*Triticum*	1997	India	OL653677	+	−	+	+
	DAOMC 236411	*Triticum*	1997	India	OL653678	+	−	+	+
	DAOMC 236412	*Triticum*	1985	Mexico	OL653679	+	−	+	+
	DAOMC 236414	*Triticum*	1986	Pakistan	OL653680	+	−	+	+
	DAOMC 236415	*Triticum*	1995	India	OL653681	+	−	+	+
	DAOMC 236416	*Triticum*	1997	Pakistan	OL653682	+	−	+	+
	DAOMC 236417	*Triticum*	1997	Pakistan	OL653683	+	−	+	+
	DAOMC 236418	*Triticum*	1996	Mexico	OL653684	+	−	+	+
	DAOMC 236419	*Triticum*	1997	India	OL653685	+	−	+	+
	DAOMC 236420	*Triticum*	1997	India	OL653686	+	−	+	+
	DAOMC 236421	*Triticum*	1997	Pakistan	OL653687	+	−	+	+
	DAOMC 238027	*Triticum*	not known	Mexico	HQ317519	+	−	+	+
	DAOMC 238045	*Triticum*	1981	Mexico	OL653699	+	−	+	+
	DAOMC 238046	*Triticum*	1991	India	OL653700	+	−	+	+
	DAOMC 238047	*Triticum*	1995	USA	HQ317581	+	−	+	+
	DAOMC 238048	*Triticum*	1997	India	OL653701	+	−	+	+
*T. walkeri*	DAOMC 236422	*Lolium*	1996	USA	OL653688	−	+	+	+
	DAOMC 236423	*Lolium*	1996	USA	OL653689	−	+	+	+
	DAOMC 238049	*Lolium*	1998	USA	OL653702	−	+	+	+
*T. asperifolia*	ATCC 90929	*Muhlenbergia*	not known	USA	OL653714	−	−	−	−
*T. brevifaciens*	CBS 121948	*Thinopyrum*	not known	Poland	OL653708	−	−	−	−
*T. bromi*	CBS 123001	*Bromus*	not known	USA	OL653706	−	−	−	−
	CBS 123002	*Bromus*	not known	USA	OL653705	−	−	−	−
	ATCC 90927	*Bromus*	not known	USA	OL653712 ^c^	−	−	−	−
	DAOMC 238034	*Bromus*	1991	USA	OL653691 ^c^	−	−	−	−
	DAOMC 238035	*Bromus*	1995	USA	OL653692 ^c^	−	−	−	−
	DAOMC 238036	*Bromus*	1991	USA	OL653693 ^c^	−	−	−	−
	ATCC 90928	*Bromus*	not known	USA	OL653713 ^d^	−	−	−	−
*T. caries*	CBS 121951	*Triticum*	not known	Sweden	OL653707	−	−	−	−
	DAOMC 238032	*Triticum*	1996	USA	HQ317579	−	−	−	−
	DAOMC 238033	*Triticum*	1996	USA	HQ317580	−	−	−	−
*T. controversa*	ATCC 42079	*Triticum*	not known	USA	OL653710 ^e^	−	−	−	−
	DAOMC 236426	*Triticum*	1998	Canada	HQ317522	−	−	−	−
	DAOMC 238052	*Triticum*	1997	Canada	OL653703 ^e^	−	−	−	−
*T. fusca*	ATCC 90926	*Vulpia*	not known	USA	OL653711	−	−	−	−
	DAOMC 238041	*Vulpia*	1996	USA	OL653696	−	−	−	−
	DAOMC 238042	*Vulpia*	1995	USA	OL653697	−	−	−	−
	DAOMC 238043	*Vulpia*	1995	USA	OL653698	−	−	−	−
	DAOMC 238053	*Vulpia*	1995	USA	OL653704	−	−	−	−
*T. goloskokovii*	CBS 122995	*Apera*	not known	USA	OL653709	−	−	−	−
*T. horrida*	DAOMC 236425 ^b^	*Oryza*	1997	USA	HQ317521	−	−	−	−
	DAOMC 238029 ^b^	*Oryza*	1996	USA	OL653690	−	−	−	−
*T. laevis*	DAOMC 238039	*Triticum*	1997	Australia	OL653694	−	−	−	−
	DAOMC 238040	*Triticum*	1997	Australia	OL653695	−	−	−	−

^a^ DAOMC: Canadian Collection of Fungal Cultures, Ottawa, ON, Canada; CBS: CBS-KNAW Filamentous Fungi Collection, Westerdijk Fungal Biodiversity Institute, Utrecht, The Netherlands; ATCC: American Type Culture Collection, Manassas, VA, USA The CBS cultures were received under CFIA import permit P-2013-01007; ^b^ Received as *T. barclayana*; redetermination based on host and comparison of 28S sequence data with AY818974 and AY818975 [60]; ^c^ Current name for specimen received as *T. fusca* var. *bromi-tectorum* [36]. ^d^ Current name for specimen received as *T. fusca* var. *guyotiana* [36]; ^e^ Based on forward sequence only; reverse sequence failed due to polybase region (>5xT) at 3′ end of the ITS2 spacer.

**Table 3:** Voucher numbers, provenance, year collected, ITS GenBank accession numbers and assay validation results for the field-collected environmental *Tilletia* specimens used in this study.

Name	Voucher No. ^a^	Year Collected	Provenance	ITS GenBank Accession No.	TaqMan qPCR Results			
					TinOG09272 *T. indica*	TwaOG10415 *T. walkeri*	OG08220 *T. indica* and *T. walkeri*	OG01193 *T. indica* and *T. walkeri*
*T. indica*	KBW 005	1991	India	OL636488	+	−	+	+
	KBW 011	1997	India	OL636489	+	−	+	+
	KBW 012	1997	India	OL636490	+	−	+	+
	KBW 017	1981	Mexico	OL636491	+	−	+	+
	KBW 029	1991	Mexico	OL636492	+	−	+	+
	KBW 038	1984	USA	OL636493	+	−	+	+
	KBW 039	1985	Pakistan	OL636498	+	−	+	+
	KBW 042	2000	S. Africa	OL636494	+	−	+	+
	KBW 047	1995	USA	OL636495	+	−	+	+
	KBW 050	1996	USA	OL636496	+	−	+	+
	KBW 132	1996	India	OL636497	+	−	+	+
*T. brevifaciens*	TBY 001	1995	USA	OL653669 ^b^	−	−	−	−
*T. bromi*	TBH 003	1990	USA	OL653673 ^b^	−	−	−	−
	TBH 004	1990	USA	OL653671 ^b^	−	−	−	−
*T. caries*	TCT 030	2006	USA	OL636486	−	−	−	−
*T. controversa*	TCK 010	1990	USA	OL653668 ^b^	−	−	−	−
*T. horrida*	THT 003	1990	USA	None ^c^	−	−	−	−
	THT 007	1993	Philippines	OL653672 ^b,c^	−	−	−	−
	THT 009	1995	USA	None ^c^	−	−	−	−
*T. laevis*	TLT 012	1990	USA	OL636487	−	−	−	−
*T. pallida*	TPF 001	1995	USA	OL653670 ^b,d^	−	−	−	−

^a^ Received from the United States Department of Agriculture under CFIA import permits P-2014-03260 and P-2014-03259; ^b^ Short ITS1 sequence from PCR and sequencing using primers MK56-F and Tilletia-R; ^c^ Tested positive with Tan et al. [49] *T. horrida* assay; ^d^ No Genbank data for species; 88% (113/128) BLAST to *T. lachnagrostidis* MH231790.

**Table 4:** *Tilletia* qPCR assay primers and probes, annealing temperatures and limit of detection.

Target/Assay Name	Primer/Probe Name	Direction/Probe	Sequence 5′→3′ ^a^	Annealing Temperature (°C)
*Tilletia indica*/TinOG09272 (Nguyen et al. [43])	OG09272.Tin.F1	Forward	GAGGACCTTCAAGATCTGACAGG	56
	OG09272.Tin.R1	Reverse	CTGATGATCTTGCCCGGTTTTAC	
	OG09272.Tin.P1	Probe	56-FAM/ACACCTAGG/ZEN/CCACTCCCTATCCAGCCA/3IABkFQ	
*T. walkeri*/TwaOG10415 (Nguyen et al. [43])	OG10415.Twa.F1	Forward	TCAACTACTTCGACTCCTCCTCC	56
	OG10415.Twa.R1	Reverse	GCGACACCATCCTTAGTTGTGTA	
	OG10415.Twa.P1	Probe	56-FAM/CTTCCGTGA/ZEN/TCCCGTCAACGTCGGACT/3IABkFQ	
*T. indica* & *T. walkeri* complex/OG01193 (this study)	OG01193.Tin.Twa.F2	Forward	CAAAGGTCAGCTGCGAGGC	68
	OG01193.Tin.Twa.R2	Reverse	TTCGCCTTTCCTTCCCTTAAGAG	
	OG01193.Tin.Twa.P2	Probe	56-FAM/ATTACGGCG/ZEN/ACGTACAGCTTCTACCGACTTA/3IABkFQ	
*T. indica* & *T. walkeri* complex/OG08220 (this study)	OG08220.Tin.Twa.F1	Forward	ACTGTGACCCTAAACGGTGTGA	60
	OG08220.Tin.Twa.R1	Reverse	TGCTCTGGAGGAGCCGGA	
	OG08220.Tin.Twa.P2	Probe	56-FAM/TCCGCTCAA/ZEN/ATCAACAACTCGGGTAACCCGGT/3IABkFQ	

^a^ Obtained from IDT (Integrated DNA Technologies, Coralville, IA, USA; https://www.idtdna.com, accessed on 8 November 2021).

**Table 5:** Reference genomes used to design qPCR assays. Table adapted from Nguyen et al. [43].

*Tilletia* Species	Voucher ^a^	NCBI BioBroject	NCBI SRA
*caries*	DAOMC 238032	PRJNA317434	SRR3337315 and SRR3337316
*controversa*	DAOMC 234426	PRJNA317433	SRR3337317, SRR3337319, SRR3337313, SRR6305999, SRR6306000, SRR6305997 and SRR6305998
*controversa*	DAOMC 238052	PRJNA393324	SRR6305452
*indica*	DAOMC 236408	PRJNA393304	SRR6305449
*indica*	DAOMC 236414	PRJNA393317	SRR6305448
*indica*	DAOMC 236416	PRJNA314779	SRR3286921, SRR3286931 and SRR3289824
*laevis*	ATCC 42080	PRJNA393337	SRR6305450
*laevis*	DAOMC 238040	PRJNA393335	SRR6305451
*walkeri*	DAOMC 236422	PRJNA314785	SRR3286971 and SRR3289831
*walkeri*	DAOMC 238049	PRJNA393320	SRR6305426 and SRR6305427

^a^ DAOMC: Canadian Collection of Fungal Cultures, Ottawa, ON, Canada; ATCC: American Type Culture Collection, Manassas, VA, USA.

## Data Availability

Reference ITS sequences for the voucher material used in this study are deposited in GenBank (Table 2 and Table 3, and Supplementary Table S1: OL636509-OL636519 and OL712411-OL712415).

## Supplementary Materials

The following are available online at https://www.mdpi.com/article/10.3390/biology10121295/s1, Supplementary Table S1: Non-*Tilletia* wheat or grass pathogens and wheat (host plant) against which the qPCR assays were tested. Supplementary Data S1: A. Sequence alignment of the genic region OG08220 for *Tilletia walkeri* and *T. indica* isolates sequenced by Nguyen et al. [43]; B. Sequence alignment of the isolates sequenced by Nguyen et al. [43] presenting part of the region OG01193 unique to *Tilletia walkeri* and *T. indica* used for the qPCR assay design. References [68,69,70] are cited in the supplementary materials.

## Appendix A. PCR Protocols for Temperature Optimization

For assays TinOG09272 and TwaOG10415, the 25 µL reaction comprised 0.48 µM of each forward and reverse respective primer (Table 4), and one unit of 2 × TaqMan Eco Master Mix. The 3-step cycling conditions, run on a ViiA 7 Real-Time PCR System, were 5 min at 95 °C followed by 50 cycles consisting of 15 s at 95 °C, 30 s at 56 °C and 72 °C for 30 s. PCR products were evaluated on a Qiaxcel Advanced instrument (QIAGEN) using a QIAxcel DNA High Resolution Kit (1200) (QIAGEN) and following the user’s manual for the OM500 method.

For assays OG01193 and OG08220, the PCR reactions prepared were similar to those described in Malloch et al. [63] (see Section 2.1) and run on an Eppendorf Mastercycler pro S. The 10 µL reactions comprised 0.08 µM of each forward and reverse primer, 0.5 × Titanium Taq DNA polymerase (Takara), 1 × Titanium Taq buffer (Takara), 0.1 mM dNTPs, 1 µL of stock DNA and PCR-grade water for the remaining volume. Cycling conditions were 3 min at 95 °C; 45 cycles for 1 min at 95 °C, 1 min 30 s at 52, 54.6, 56, 58.5, 60, 62, and 64.6 °C for OG01193, and 52, 54.6, 56, and 58.5 °C for OG08220, and 2 min at 72 °C for each cycle; and 8 min at 72 °C.

Once amplification was confirmed at lower temperatures with the Titanium Taq polymerase, testing was pursued using the QuantiTect Probe PCR Kit at higher temperatures. The 10 µL QuantiTect reactions comprised 1 × QuantiTect Probe PCR master mix, 0.4 µM of each forward and reverse primer, 1 µL of stock DNA and PCR-grade water for the remaining volume. The reactions were performed as follows: 10 min at 95 °C; 50 cycles for 15 min at 95 °C and 30 s at 62, 64.6, 66, and 68.5 °C for OG01193, and 58.5, 60, 62, 64.6, 66, and 68.5 °C for OG08220 for each cycle; and 30 s at 40 °C. PCR products were loaded on a 1.5% agarose gel, and visualized on a Gel Doc XR+ instrument (Bio-Rad, Hercules, California, CA, USA) (data not shown).
