# Supplementary material for: Four In Silico Designed and Validated qPCR Assays to Detect and Discriminate Tilletia indica and T. walkeri, Individually or as a Complex
Source: Biology (Basel). 2021 Dec 8;10(12):1295. doi: 10.3390/biology10121295 (PMC8698337; doi:10.3390/biology10121295)
Supplement: Supplementary file 1 [file biology-10-01295-s001.zip › Supplementary Data S1.pdf]

Supplementary Data S1. A. Sequence alignment of the genic region OG08220 for *Tilletia walkeri* and *T. indica* isolates sequenced by Nguyen et al. [43]. Highlighted regions correspond to the qPCR primer sequences (grey) and the probe sequence (yellow).

```
                          241       250       260       270       280       290       300
                          |         |         |         |         |         |         |
T_walkeri_DAOMC_230849    GCTACCAACGCAACTGTGACCCTAAACGGTGTGAAGGGTAACATCAATCTCAACATCAAT
T_walkeri_DAOMC_236422    GCTGCCAACGCAACTGTGACCCTAAACGGTGTGAAGGGTAACATCAATCTCAACATCAGT
T_indica_DAOMC_236408     GCTTCCAACGCAACTGTGACCCTAAACGGTGTGAAGGGTAACATCAATCTCAACATCAGT
T_indica_DAOMC_236414     GCTACCAACGCAACTGTGACCCTAAACGGTGTGAAGGGTAACATCAATCTCAACATCAAT
T_indica_DAOMC_236416     GCTTCCAACGCAACTGTGACCCTAAACGGTGTGAAGGGTAACATCAATCTCAACATCAGT

                          301       310       320       330       340       350       360
                          |         |         |         |         |         |         |
T_walkeri_DAOMC_230849    GGTGGTTCCGCTCAAATCAACAACTCGGGTAACCCGGTCGATCCCGCCCTTCCTCTGCAA
T_walkeri_DAOMC_236422    GGTGGTTCCGCTCAAATCAACAACTCGGGTAACCCGGTCGATCCCGCCCTTCCTCTGCAA
T_indica_DAOMC_236408     GGTGGTTCCGCTCAAATCAACAACTCGGGTAACCCGGTCGATCCCGCCCTTCCTCTGCAA
T_indica_DAOMC_236414     GGTGGTTCCGCTCAAATCAACAACTCGGGTAACCCGGTCGATCCCGCCCTTCCTCTGCAA
T_indica_DAOMC_236416     GGTGGTTCCGCTCAAATCAACAACTCGGGTAACCCGGTCGATCCCGCCCTTCCTCTGCAA
                          361       370       380       390       400       410       420
                          |         |         |         |         |         |         |
T_walkeri_DAOMC_230849    GTCACGCTGCTCGAGAGCACGGTCCGGCTCCTCCAGAGCAAGGTTGACCGGATGAGTCGC
T_walkeri_DAOMC_236422    GTCACGCTGCTCGAGAGCACGGTCCGGCTCCTCCAGAGCAAGGTGGACCGGATGAGTCGC
T_indica_DAOMC_236408     GTCACGCTGCTCGAGAGCACGGTCCGGCTCCTCCAGAGCAAGGTGGACCGGATGAGTCGC
T_indica_DAOMC_236414     GTCACGCTGCTCGAGAGCACGGTCCGGCTCCTCCAGAGCAAGGTGGACCGGATGAGTCGC
T_indica_DAOMC_236416     GTCACGCTGCTCGAGAGCACGGTCCGGCTCCTCCAGAGCAAGGTGGACCGGATGAGTCGC
```

Supplementary Data S1. B. Sequence alignment of the isolates sequenced by Nguyen et al. [43] presenting part of the region OG01193 unique to *Tilletia walkeri* and *T. indica* used for the qPCR assay design. Highlighted regions correspond to the qPCR primer sequences (grey), and the probe sequence (yellow). Differences with the non-target species in the alignment are highlighted in cyan.

```
                              121       130       140       150       160       170       180
                              |         |         |         |         |         |         |
T_caries_DAOMC_238032         CTACCCGCGCGTTGAACATCTACGAAGGCCAACAACGAGGTTTCAAGTACCTCACTCCCA
T_laevis_ATCC_42080           CTACCCGCGCGTTGAACATCTACGAAGGCCAACAACGAGGTTTCAAGTACCTCACTCCCA
T_laevis_DAOMC_238040         CTACCCGCGCGTTGAACATCTACGAAGGCCAACAACGAGGTTTCAAGTACCTCACTCCCA
T_controversa_DAOMC_236426    CTACCCGCGCGTTGAACATCTACGAAGGCCAACAACGAGGTTTCAAGTACCTCACTCCCA
T_controversa_DAOMC_238052    CTACCCGCGCGTTGAACATCTACGAAGGCCAACAACGAGGTTTCAAGTACCTCACTCCCA
T_indica_DAOMC_236414         CAACTCGTGCGGTGAATCTCTGCAAAGGTCAACTGCGAGGCTTCAAGTACCTCACTCCCA
T_indica_DAOMC_236416         CAACTCGTGCGGTGAACCTCTACAAAGGTCAGCTGCGAGGCTTCAAGTACCTCACTCCCA
T_indica_DAOMC_236408         CAACTCGTGCGGTGAATCTCTACAAAGGTCAGCTGCGAGGCTTCAAGTACCTCACTCCCA
T_walkeri_DAOMC_236422        CAACTCGTGCGGTGAATCTCTACAAAGGTCAGCTGCGAGGCTTCAAGTACCTCACTCCCA
T_walkeri_DAOMC_230849        CAACTCGTGCGGTGAATCTCTACAAAGGTCAGCTGCGAGGCTTCAAGTACCTCACTCCCA

                              181       190       200       210       220       230       240
                              |         |         |         |         |         |         |
T_caries_DAOMC_238032         AACTTCGTCTCGATCCTCGTGATCTGCTGTACGATTCCGGCGATGGGCGACACCAGCTAC
T_laevis_ATCC_42080           AACTTCGTCTCGATCCTCGTGATCTGCTGTACGATTCCGGCGATGGGCGACACCAGCTAC
T_laevis_DAOMC_238040         AACTTCGTCTCGATCCTCGTGATCTGCTGTACGATTCCGGCGATGGGCGACACCAGCTAC
T_controversa_DAOMC_236426    AACTTCGTCTCGATCCTCGTGATCTGCTGTACGATTCCGGCGATGGGCGACACCAGCTAC
T_controversa_DAOMC_238052    AACTTCGTCTCGATCCTCGTGATCTGCTGTACGATTCCGGCGATGGGCGACACCAGCTAC
T_indica_DAOMC_236414         AGATTCGTCTGGACCCTCACGATCAGCTATATGATTACGGCGACGTACA---------GC
T_indica_DAOMC_236416         AGATTCGTCTGGACCCTCACGATCAGCTATATGATTACGGCGACGTACA---------GC
T_indica_DAOMC_236408         AGATTCGTCTGGACCCTCACGATCAGCTATATGATTACGGCGACGTACA---------GC
T_walkeri_DAOMC_236422        AAATTCGTCTGGACCCTCACGATCAGCTATACGATTACGGCGACGTACA---------GC
T_walkeri_DAOMC_230849        AAATTCGTCTGGACCCTCACGATCAGCTATACGATTACGGCGACGTACA---------GC

                              241       250       260       270       280       290       300
                              |         |         |         |         |         |         |
T_caries_DAOMC_238032         TGCTGCCGACGCATATCCACTTCATATGCTCGCCGGAGCGAGCGAGATTGATCATTGACG
T_laevis_ATCC_42080           TGCTGCCGACGCATATCCACTTCATATGCTCGCCGGAGCGAGCGAGATTGATCATTGACG
T_laevis_DAOMC_238040         TGCTGCCGACGCATATCCACTTCATATGCTCGCCGGAGCGAGCGAGATTGATCATTGACG
T_controversa_DAOMC_236426    TGCTGCCGACGCATATCCACTTCATATGCTCGCCGGAGCGAGCGAGATTGATCATTGACG
T_controversa_DAOMC_238052    TGCTGCCGACGCATATCCACTTCATATGCTCGCCGGAGCGAGCGAGATTGATCATTGACG
T_indica_DAOMC_236414         TTCTACCGACTTATATCCACTTCGTCTGCTCGCCTGAGCGGGCGAGGCTGATCGTTGACG
T_indica_DAOMC_236416         TTCTACCGACTTATATCCACTTCGTCTGCTCGCCTGAGCGGGCGAGGCTGATCGTTGACG
T_indica_DAOMC_236408         TTCTACCGACTTATATCCACTTCGTCTGCTCGCCTGAGCGGGCGAGGTTGATCGTTGACG
T_walkeri_DAOMC_236422        TTCTACCGACTTATATCCACTTCGTCTGCTCGCCTGAGCGGGCGAGGTTGATCGTTGACG
T_walkeri_DAOMC_230849        TTCTACCGACTTATATCCACTTCGTCTGCTCGCCTGAGCGGGCGAGGTTGATCGTTGACG

                              301       310       320       330       340       350       360
                              |         |         |         |         |         |         |
T_caries_DAOMC_238032         AGATTGAGGAGTTGCTGAGGGATCGGACTGCTACGAATGCGGGGAGGCCGGCGCTGATCT
T_laevis_ATCC_42080           AGATTGAGGAGTTGCTGAGGGATCGGACTGCTACGAATGCGGGGAGGCCGGCGCTGATCT
T_laevis_DAOMC_238040         AGATTGAGGAGTTGCTGAGGGATCGGACTGCTACGAATGCGGGGAGGCCGGCGCTGATCT
T_controversa_DAOMC_236426    AGATTGAGGAGTTGCTGAGGGATCGGACTGCTACGAATGCGGGGAGGCCGGCGCTGATCT
T_controversa_DAOMC_238052    AGATTGAGGAGTTGCTGAGGGATCGGACTGCTACGAATGCGGGGAGGCCGGCGCTGATCT
T_indica_DAOMC_236414         AGGTCGAGGAGCTCTTAAGGGAAGGAAAGGCGAAGAATAAGGAGAGGCCGGCCTTGATCT
T_indica_DAOMC_236416         AAGTCGAGGGGCTCTTAAGGGAAGGAAAGGCGAAGAATAAGGAAAGGCCGGCCTTGATCT
T_indica_DAOMC_236408         AAGTCGAGGAGCTCTTAAGGGAAGGAAAGGCGAAAAATAAGGAAAGGCCGGCCTTGATCT
T_walkeri_DAOMC_236422        AAGTCGAGGAGCTCTTAAGGGAAGGAAAGGCGAAAAATAAGGAAAGGCCGGCCTTGATCT
T_walkeri_DAOMC_230849        AAGTTGAGGAGCTCTTAAGGGAAGGAAAGGCGAAAAATAAGGAAAGGCCGGCCTTGATCT
```
