# Supplementary material for: Four In Silico Designed and Validated qPCR Assays to Detect and Discriminate Tilletia indica and T. walkeri, Individually or as a Complex
Source: Biology (Basel). 2021 Dec 8;10(12):1295. doi: 10.3390/biology10121295 (PMC8698337; doi:10.3390/biology10121295)
Supplement: Supplementary file 1 [file biology-10-01295-s001.zip › Supplementary Table S1.pdf]

Supplementary Table S1. Non-*Tilletia* wheat or grass pathogens and wheat (host plant) against which the qPCR assays were tested.

Species	Voucher [a]	Disease associated	ITS GenBank Acc. No.	Reference
Alternaria alternata	DAOMC 234879	Black point / smudge	OL636518	this study
Aspergillus foetidus	KAS3540	Grain-storage molds	OL636513	this study
Bipolaris sorokiniana	DAOMC 226217	Spot blotch	OL636509	this study
Blumeria graminis	DAOM 38670	Powdery mildew	OL712411	this study
Cladosporium allicinum	KAS4624	Black (sooty) head mold	OL636514	this study
Cladosporium herbarum	JB2212	Black (sooty) head mold	OL636517	this study
Didymella glomerata	JB2038	Leaf spot / leaf blight	OL636515	this study
Didymella glomerata	JB2113	Leaf spot / leaf blight	OL636516	this study
Fusarium graminearum	SH1008	Fusarium head blight (Scab)	OL636519	this study
Parastagonospora nodorum	DAOMC 226222	Glume blotch	OL636510	this study
Penicillium verrucosum	KAS6850	Grain-storage molds	OL636512	this study
Puccinia graminis	RS1924	Stem rust	OL712413	this study
Puccinia pseudostriiformis	PUR N5368	Stripe rust	HM057133	[68]
Puccinia pseudostriiformis	PUR 59844	Stripe rust	MT965634	[69]
Puccinia striiformis	PUR 66275	Stripe rust	HM057112	[68]
Puccinia striiformis	DAOM 240069	Stripe rust	HM057118	[68]
Puccinia striiformis	BPI 193871	Stripe rust	HM057135	[68]
Puccinia striiformoides	PUR N5378	Stripe rust	HM057110	[68]
Puccinia striiformoides	K(M): 108141	Stripe rust	HM057125	[68]
Puccinia striiformoides	PUR N5374	Stripe rust	HM057111	[68]
Puccinia triticina	DAOM 240852	Leaf rust	HQ012456	[70]
Pyrenophora tritici-repentis	DAOMC 226213	Tan spot	JN943670	Fungal Barcoding Consortium
Septoria glycines	DAOMC 226244	Brown spot	OL636511	this study
Triticum sp.	RS2089	None; uninfected wheat host	OL712415	this study
Urocystis tritici	RS2070	Flag smut of wheat	OL712414	this study
Ustilago nuda	RS220	Loose smut of barley	OL712412	this study

[a] **DAOM**: Canadian National Mycological Herbarium, Ottawa, ON, Canada; **DAOMC**: Canadian Collection of Fungal Cultures, Ottawa, ON, Canada; **PUR**: Arthur Herbarium, Purdue University, West Lafayette, IN, USA; **BPI**: US National Fungus Collections, USDA-ARS, Beltsville, MD, USA.; **K(M)**: Royal Botanic Gardens, Kew, UK; **KAS, JB, RS, SH**: AAFC internal DNA ID identifiers.
